# Chaperonin-Containing TCP1 Subunit 5 Protects Against the Effect of Mer Receptor Tyrosine Kinase Knockdown in Retinal Pigment Epithelial Cells by Interacting With Filamentous Actin and Activating the LIM-Kinase 1/Cofilin Pathway

**DOI:** 10.3389/fmed.2022.861371

**Published:** 2022-04-13

**Authors:** Lujia Feng, Haichun Li, Yong Du, Ting Zhang, Yingting Zhu, Zhidong Li, Ling Zhao, Xing Wang, Gongpei Wang, Linbin Zhou, Zhaorong Jiang, Zheng Liu, Zhancong Ou, Yuwen Wen, Yehong Zhuo

**Affiliations:** ^1^State Key Laboratory of Ophthalmology, Zhongshan Ophthalmic Center, Guangdong Provincial Key Laboratory of Ophthalmology and Visual Science, Guangdong Provincial Clinical Research Center for Ocular Diseases, Sun Yat-sen University, Guangzhou, China; ^2^Guizhou Provincial People’s Hospital, Guizhou University, Guiyang, China; ^3^Ophthalmology Department of Zhuhai Integrated Traditional Chinese and Western Medicine Hospital, Zhuhai, China

**Keywords:** retinitis pigmentosa, *MERTK*, CCT5, f-actin, phagocytosis

## Abstract

Retinitis pigmentosa (RP), characterized by the gradual loss of rod and cone photoreceptors that eventually leads to blindness, is the most common inherited retinal disorder, affecting more than 2.5 million people worldwide. However, the underlying pathogenesis of RP remains unclear and there is no effective cure for RP. Mutations in the Mer receptor tyrosine kinase (*MERTK*) gene induce the phagocytic dysfunction of retinal pigment epithelium (RPE) cells, leading to RP. Studies have indicated that filamentous actin (F-actin)—which is regulated by chaperonin-containing TCP1 subunit 5 (CCT5)—plays a vital role in phagocytosis in RPE cells. However, whether CCT5/F-actin signaling is involved in MERTK-associated RP remains largely unknown. In the present study, we specifically knocked down *MERTK* and *CCT5* through siRNA transfection and examined the expression of CCT5 and F-actin in human primary RPE (HsRPE) cells. We found that MERTK downregulation inhibited cell proliferation, migration, and phagocytic function; significantly decreased the expression of F-actin; and disrupted the regular arrangement of F-actin. Importantly, our findings firstly indicate that CCT5 interacts with F-actin and is inhibited by *MERTK* siRNA in HsRPE cells. Upregulating CCT5 using *CCT5*-specific lentiviral vectors (*CCT5*-Le) rescued the cell proliferation, migration, and phagocytic function of HsRPE cells under the *MERTK* knockdown condition by increasing the expression of F-actin and restoring its regular arrangement *via* the LIMK1/cofilin, but not the SSH1/cofilin, pathway. In conclusion, CCT5 protects against the effect of *MERTK* knockdown in HsRPE cells and demonstrates the potential for effective treatment of MERTK-associated RP.

## Introduction

Retinitis pigmentosa (RP) is the most common inherited retinal disease that affected tens of millions of people worldwide and is caused by a series of gene mutations that eventually lead to progressive retinal degeneration (1). As the most common inherited retinal disease, RP affects more than 2.5 million people worldwide (2, 3). However, the pathogenesis of RP remains unclear, and there is currently no effective treatment (4). Studies have shown that the Mer receptor tyrosine kinase (MERTK) gene mutation can lead to RP in humans (5). In the Royal College of Surgeons (RCS) rat, which is a typical animal model of human autosomal recessive inherited RP, the disease was found to be caused by the *Mertk* gene mutation (6). The *MERTK* gene encodes the Mer receptor tyrosine kinase, which belongs to the TAM receptor kinase family and participates in the phagocytic process (5). Retinal pigment epithelium (RPE) can constantly phagocytize the shed photoreceptor outer segment (POS), which plays a vital role in maintaining retinal homeostasis, as POS renewal is essential for visual function (7). The phagocytic dysfunction of RPE is an important aspect of the pathogenesis of RP (8). According to previous studies, a mutation in the *MERTK* gene leads to the phagocytic dysfunction of RPE cells, which is responsible for MERTK-associated RP (5). However, the mechanism underlying the *MERTK* gene mutation leading to phagocytic dysfunction remains largely unknown.

The process of POS phagocytosis is based on the rigorous control of the distribution and expression of the actin cytoskeleton (9). Actin, as a major component of the cytoskeleton, plays a vital role in the POS phagocytosis process. A previous study showed alphavbeta5 binding POS which is the first step of the POS phagocytosis process is required actin (10). However, nearly decades after the phenomenon was first described, much remains unknown regarding the role of the actin cytoskeleton in this process. Recent studies have emphasized that the rearrangement of RPE cytoskeletal filamentous actin (F-actin) is essential for POS internalization (11). The formation of phagocytic cups *via* the early recruitment of F-actin lays the foundation for phagocytosis, which means any factor that obstructs the expression of F-actin or disrupts the arrangement of F-actin will lead to phagocytic dysfunction, as the phagocytic cup formed by the orderly aggregation of F-actin combined with POS is the key to phagocytosis initiation (12). Previous studies demonstrated that the F-actin arrangement is abnormal in RPE cells with low phagocytotic activity (13). Recent studies also indicated that phagocytes formed by the recruitment of F-actin and the binding of POS must be combined with MERTK (14). In other words, the *MERTK* gene mutation may impair the recruitment of F-actin. However, the involvement of abnormal epithelial F-actin cytoskeleton in MERTK-associated RP remains largely unknown.

The TCP-1 cyclic complex, also known as the chaperonin-containing TCP-1 (CCT), is composed of eight parallel subunits (CCT1–8) (15–17). Assisted by ATP binding and hydrolysis, the main function of CCT is to help refold misfolded or unfolded proteins. Between 5% and 10% of the proteins in mammalian cells interact with CCT (18). In S. cerevisiae, CCT deficiency mutations lead to death, demonstrating the importance of CCT function (19). Actin is one of the main substrates of CCT; however, the exact mechanism by which CCT promotes actin folding is not yet understood (20, 21). A recent study found that CCT was required for efficient actin myofilament assembly (22) and that CCT5-specific ATP binding was required for efficient actin folding (23). Additionally, CCT5 controls lysosome biogenesis *via* the actin cytoskeleton (24). The actin/CCT5 pathway is implicated in multiple diseases—including hereditary sensory neuropathies (23), legionella pneumophila infection (25), muscle atrophy (26), and Alzheimer’s disease (24)—suggesting that CCT5 is associated with various cellular physiological processes in different tissues. Nonetheless, whether the F-actin/CCT5 pathway plays a vital role in MERTK-associated RP remains to be elucidated. Therefore, the present study explored the relationship between CCT5, F-actin, and RP, and the potential molecular mechanism underlying this disease.

## Results

### Mer Receptor Tyrosine Kinase siRNA Inhibited Cell Proliferation and Induced Morphological Changes and Phagocytic Dysfunction in Human Primary RPE Cells

**FIGURE 1:**
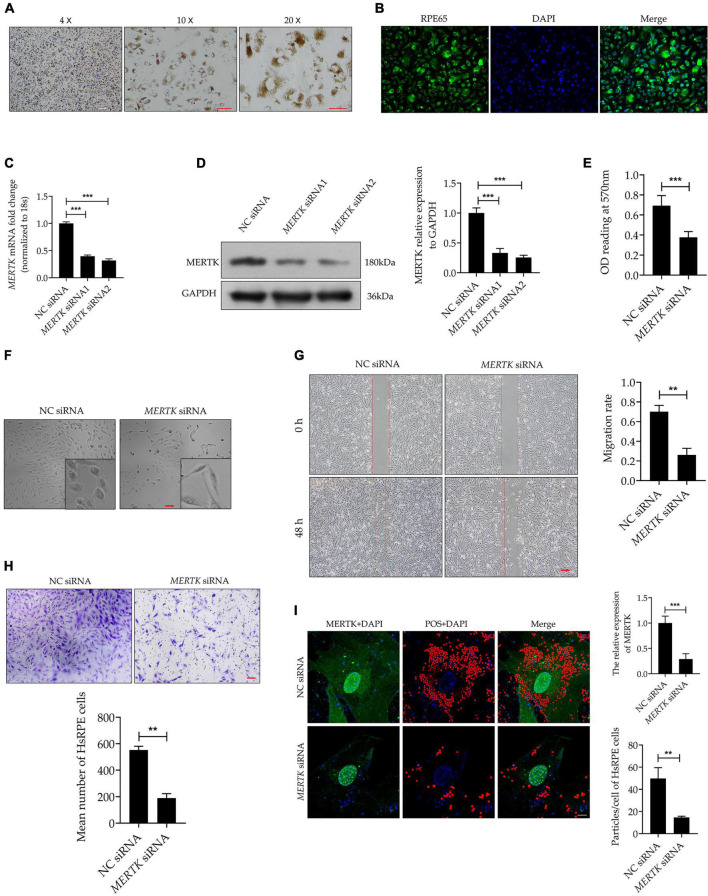
*MERTK* siRNA inhibited cell proliferation and induced morphological changes and phagocytic dysfunction in HsRPE cells. **(A)** The primary cultured HsRPE cells were observed with an inverted microscope at different magnifications (4X, 10X, and 20X). Scale bars: white bar 100 μm; red bars 10 μm. **(B)** The HsRPE cells were identified by immunofluorescence staining with RPE65 antibody. Scale bars: 50 μm. **(C)** HsRPE cells were treated with NC, *MERTK* siRNA1, or *MERTK* siRNA2 (20 nM) for 24 h, and the expression of *MERTK* was analyzed by RT-PCR. **(D)** HsRPE cells were treated with NC, *MERTK* siRNA1, or *MERTK* siRNA2 (20 nM) for 48 h, and *MERTK* expression was determined by Western blotting. **(E)** HsRPE cells were treated with NC or *MERTK* siRNA2 (*MERTK* siRNA) for 48 h, and cell proliferation was analyzed by CCK8. **(F)** HsRPE cells were treated the same as in (E), and cell morphology was observed with an inverted microscope. Scale bars: 100 μm. **(G)** HsRPE cells were treated the same as in (E), and wound-healing assays were performed to assess the wound-healing capabilities of HsRPE cells. Scale bars: 200 μm. **(H)** HsRPE cells were treated the same as in (E), and Transwell assays were performed to evaluate the migration activity of HsRPE cells (purple). Scale bars: 100 μm. **(I)** HsRPE cells were treated the same as in (E), and their phagocytic ability was examined using phagocytosis assays. The red dots represent the particles and the green dye represents the *MERTK* protein. Scale bars: 5 μm. **P < 0.01; *** P < 0.001.

On the 7 days after the HsRPE cells were extracted and cultured in DMEM medium, we observed cultured cells with an inverted phase-contrast microscope, we found each cell contains abundant brownish-yellow pigments which is the characterization of the HsRPE cell. This result suggests that we’re extracting the HsRPE cells, not other nerve cells, fibroblasts, blood vessel cells, etc (Figure 1A). To further verify that the cells we extracted were HsRPE cells, we stained the cultured cells with RPE65, an RPE cell-specific protein. The result showed every cultured cell is stained with RPE65 (green) (Figure 1B). These results indicate that we have successfully extracted and cultured HsRPE cells. To establish a model of MERTK-associated RP *in vitro*, *MERTK* siRNA was applied to HsRPE cells. To test the target-specific efficacy of the *MERTK* siRNA, two types of *MERTK* siRNA (*MERTK* siRNA1 and *MERTK* siRNA2) were used. Both the mRNA and protein expression of MERTK in the siRNA groups were significantly reduced compared with those in the negative control (NC) siRNA groups (Figures 1C,D). Since *MERTK* siRNA2 more effectively downregulated MERTK than *MERTK* siRNA1, we applied *MERTK* siRNA2 in the subsequent experiments. A Cell Counting Kit-8 (CCK8) assay was used to determine the viability of HsRPE cells. Compared with the NC siRNA group, the group with knocked down MERTK exhibited suppressed cell proliferation, as the optical density (OD) value of the NC siRNA group was higher than that of the *MERTK* siRNA group (Figure 1E). We then observed the changes in cell morphology using an inverted microscope and found that the cellular morphologies of the HsRPE cells were very different between the NC siRNA group and the *MERTK* siRNA group. The cells of the NC siRNA group had a typical polygonal appearance; however, the *MERTK* siRNA-transfected cells became elongated in shape (Figure 1F). Wound-healing assays and Transwell migration assays were performed to assess the wound-healing ability and migration of HsRPE cells. The results demonstrated that the number of migrated HsRPE cells and their migration distance was significantly decreased in the *MERTK* siRNA group compared with the NC siRNA group (Figures 1G,H). We performed the phagocytosis assay combined with the immunofluorescence assay to evaluate the effect of *MERTK* siRNA on the phagocytic function of HsRPE cells. For the phagocytosis assay, Nile red-labeled particles and MERTK immunofluorescent staining were used. In the NC siRNA group, the expression of MerTK (green) is significantly higher than MERTK siRNA group, and massive red particles were phagocytized into the HsRPE cells, while the number of red particles that were phagocytized into the HsRPE cells was significantly decreased in the *MERTK* siRNA group (Figure 1I). These results indicate that *MERTK*-specific siRNA inhibited cell proliferation and induced morphological changes and phagocytic dysfunction in HsRPE cells.

### Mer Receptor Tyrosine Kinase siRNA Downregulated the Expression of Chaperonin-Containing TCP1 Subunit 5, Which Interacted and Co-localized With Filamentous Actin in Human Primary RPE Cells

**FIGURE 2:**
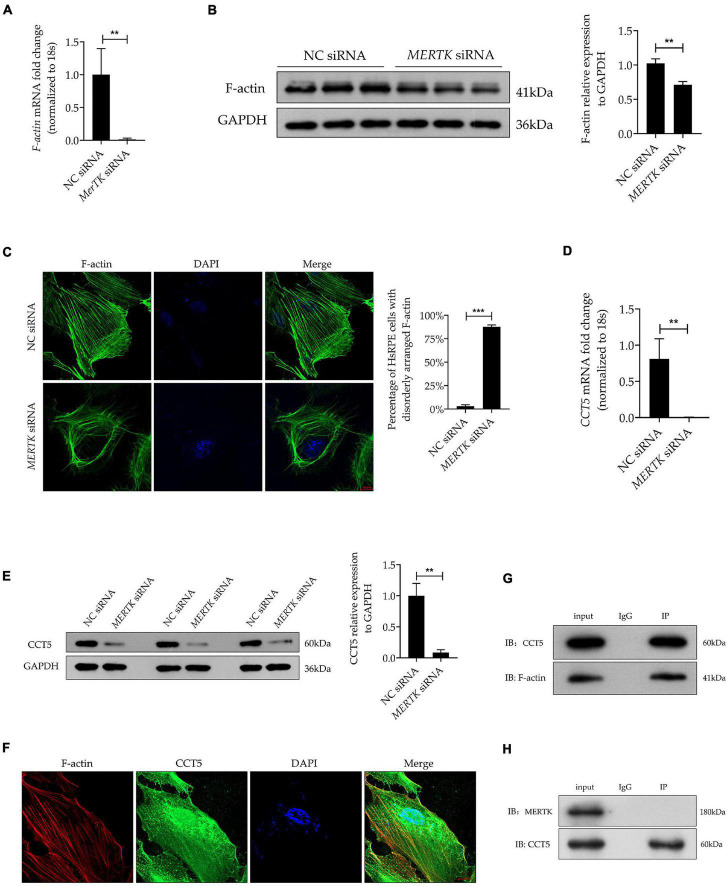
*MERTK* siRNA downregulated the expression of CCT5, which interacted and co-localized with F-actin in HsRPE cells. **(A)** HsRPE cells were treated with NC or *MERTK* siRNA for 24 h, and the expression of *F-actin* was analyzed by RT-PCR. **(B)** HsRPE cells were treated with NC or *MERTK* siRNA for 48 h, and the expression of F-actin was analyzed by Western blotting. **(C)** HsRPE cells were treated the same as in **(B)**, and the distribution and organization of F-actin (green) were detected by immunofluorescence analysis. Scale bars: 10 μm. **(D)** HsRPE cells were treated the same as in (A), and*CCT5* expression was analyzed by RT-PCR. **(E)** HsRPE cells were treated the same as in **(B)**, and CCT5 expression was analyzed by Western blotting. **(F)** The co-localization (yellow) of F-actin (red) and CCT5 (green) in HsRPE cells was assessed by immunolocalization analysis. Scale bars: 10 μm. **(G)** The binding between F-actin and CCT5 in HsRPE cells was verified by co-immunoprecipitation. **(H)** The binding between MERTK and CCT5 in HsRPE cells was verified by co-immunoprecipitation. **P < 0.01; *** *P* < 0.001.

Filamentous actin plays a vital role in phagocytosis. To determine whether F-actin was involved in MERTK-associated RP, we detected F-actin by quantitative real-time PCR (RT-PCR) and Western blot assays separately. Both the RT-PCR and Western blot assays demonstrated that *MERTK* siRNA significantly inhibited F-actin at both the mRNA and protein levels, as compared with the NC group (Figures 2A,B). Furthermore, to detect the distribution and arrangement of F-actin, we performed the immunofluorescence analysis. The results indicated that F-actin demonstrated a highly regular radial distribution of filamentous order in the NC group. However, in the *MERTK* siRNA group, the F-actin clumped together in a disorderly arrangement around the nucleus (Figure 2C). CCT5 plays an important role in actin regulation; therefore, to determine whether CCT5 was involved in the regulation of F-actin in MERTK-associated RP, CCT5 expression at the mRNA and protein levels was assessed. The RT-PCR results indicated that the downregulation of MERTK significantly suppressed the expression of *CCT5* at the mRNA level, compared with that in the NC group, in HsRPE cells (Figure 2D). In line with the mRNA-level results, the Western blotting results demonstrated that CCT5 expression was significantly suppressed by *MERTK* siRNA in HsRPE cells (Figure 2E). To analyze whether CCT5 and F-actin directly interacted, we performed immunolocalization and co-immunoprecipitation (Co-IP) assays. Immunofluorescence analysis demonstrated that CCT5 and F-actin co-localized in the cytoplasm of HsRPE cells (Figure 2F), and Co-IP analysis demonstrated that CCT5 proteins could directly bind with F-actin proteins, but could not directly bind with MERTK proteins in HsRPE cells (Figures 2G,H). These results indicate that CCT5 interacts and co-localizes with F-actin but not MERTK and that *MERTK* siRNA can decrease F-actin expression *via* the regulation of CCT5 expression in HsRPE cells.

### Upregulation of Chaperonin-Containing TCP1 Subunit 5 Expression Recovered the Morphology and Migration Function Destroyed by Mer Receptor Tyrosine Kinase siRNA in Human Primary RPE Cells

**FIGURE 3:**
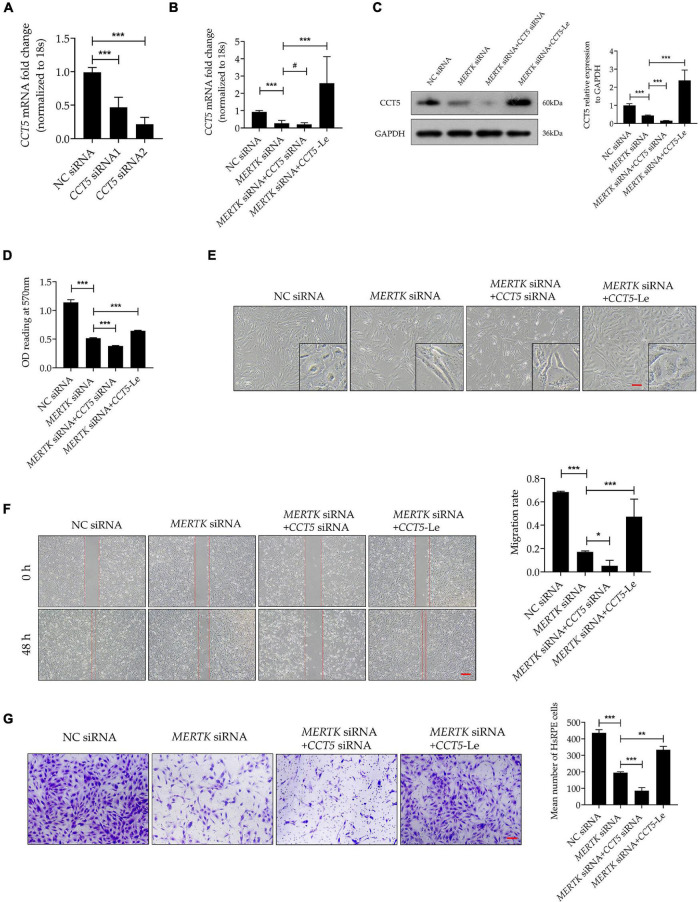
Upregulation of CCT5 expression recovered the morphology and migration function disrupted by *MERTK* siRNA in HsRPE cells. **(A)** HsRPE cells were treated with NC, *CCT5* siRNA1, or *CCT5* siRNA2 (20 nM) for 24 h, and *CCT5* expression was analyzed by RT-PCR. **(B)** HsRPE cells were treated with or without *CCT5* siRNA or *CCT5*-specific lentiviral vectors (*CCT5*-Le) after exposure to NC or *MERTK* siRNA for 24 h, and the expression of *CCT5* was analyzed by RT-PCR. **(C)** HsRPE cells were treated with or without *CCT5* siRNA or *CCT5*-specific lentiviral vectors (*CCT5*-Le) after exposure to NC or *MERTK* siRNA for 48 h, and CCT5 expression was analyzed by Western blotting. **(D)** HsRPE cells were treated the same as in (C), and cell proliferation was determined by CCK8 assay. **(E)** HsRPE cells were treated the same as in **(C)**, and morphological changes were observed using an inverted microscope. Scale bars: 100 μm. **(F)** HsRPE cells were treated the same as in **(C)**, and wound-healing assays were performed to assess the wound-healing capabilities of HsRPE cells. Scale bars: 200 μm. **(G)** HsRPE cells were treated the same as in **(C)**, and Transwell assays were performed to evaluate the migration activity of HsRPE cells (purple). Scale bars: 100 μm. # *P* > 0.05; *P < 0.05; **P < 0.01; *** *P* < 0.001.

For a better insight into the involvement of CCT5 in RP, *CCT5*-specific siRNA and lentiviral vectors were used to downregulate and upregulate CCT5 expression separately. We tested two siRNAs (*CCT5* siRNA1 and *CCT5* siRNA2) and selected *CCT5* siRNA2, showing the best downregulation efficiency, for the subsequent experiments (Figure 3A). After the *CCT5* siRNA was transfected into HsRPE cells, the expression of CCT5 was significantly downregulated. On the other hand, treatment with *CCT5*-specific lentiviral vectors (*CCT5*-Le) significantly upregulated the expression of CCT5, compared with that in the *MERTK* siRNA group, at both the mRNA and protein levels (Figures 3B,C). CCK8 assays were used to detect the viability of HsRPE cells. Compared with the *MERTK* siRNA group, the *MERTK* siRNA + *CCT5* siRNA group demonstrated significant further suppression of cell proliferation, as the optical density (OD) value of the *MERTK* siRNA + *CCT5* siRNA group was lower than that of the *MERTK* siRNA groups. However, the viability of HsRPE cells in the *MERTK* siRNA + *CCT5*-Le group was significantly higher than that seen in the *MERTK* siRNA group (Figure 3D). These results demonstrate that the upregulation of CCT5 expression enhanced the viability of HsRPE cells, which was inhibited by *MERTK* siRNA. Next, we observed changes in cell morphology using an inverted microscope. In the *MERTK* siRNA + *CCT5* siRNA group, the shape of the HsRPE cells became more elongated than in the *MERTK* siRNA group. However, in the *MERTK* siRNA + *CCT5*-Le group, the shape of the HsRPE cells became typically polygonal, as in the NC group (Figure 3E). These results indicate that upregulating CCT5 expression restored the morphological dysfunction induced by *MERTK* siRNA. Wound-healing and Transwell-migration assays demonstrated that the migration distance and the number of migrated HsRPE cells were significantly suppressed in the *MERTK* siRNA group compared with the control group and that *CCT5* siRNA further suppressed these parameters. However, *CCT5*-Le significantly increased the migration distance and the number of migrated HsRPE cells, which were suppressed by *MERTK* siRNA (Figures 3F,G). These results indicate that upregulation of CCT5 expression restored the morphology and migration function, which were disrupted in HsRPE cells with *MERTK* siRNA.

### Upregulating Chaperonin-Containing TCP1 Subunit 5 Expression Rescued the Phagocytic Function Disrupted by Mer Receptor Tyrosine Kinase siRNA by Restoring the Expression and Organization of Filamentous Actin in Human Primary RPE Cells

**FIGURE 4:**
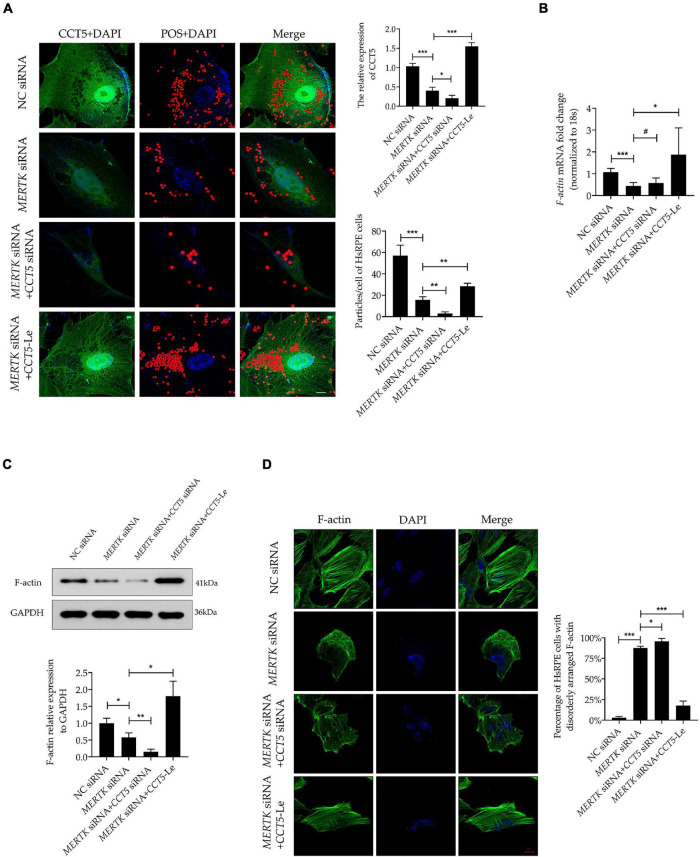
CCT5 rescued the phagocytic function disrupted by *MERTK* siRNA by restoring the expression and organization of F-actin in HsRPE cells. **(A)** HsRPE cells were treated with or without *CCT5* siRNA or *CCT5*-Le after exposure to NC or *MERTK* siRNA for 48 h, and the phagocytic ability of HsRPE cells was examined by phagocytosis assay. The red dots represent the particles and the green dye represents the CCT5 protein. Scale bars: 5 μm. **(B)** HsRPE cells were treated with or without *CCT5* siRNA or *CCT5*-Le after exposure to NC or *MERTK* siRNA for 24 h, and *F-actin* expression was analyzed by RT-PCR. **(C)** HsRPE cells were treated the same as in (A), and F-actin expression was analyzed by Western blotting. **(D)** HsRPE cells were treated the same as in (A), and the distribution and organization of F-actin (green) were detected by immunofluorescence analysis. Scale bars: 10 μm. # *P* > 0.05; *P < 0.05; **P < 0.01; *** *P* < 0.001.

To investigate the function of *CCT5*-Le in the phagocytic process of HsRPE cells, we performed the phagocytosis assay combined with the immunofluorescence assay. The *MERTK* siRNA group exhibited a significantly decreased expression of CCT5 (green) and a significantly decreased quantity of red particles that were phagocytized into the HsRPE cells, as compared with the NC siRNA group. In the *MERTK* siRNA + *CCT5* siRNA group, the expression of CCT5 was further decreased and the number of red particles phagocytized into HsRPE cells was further reduced compared with that in the *MERTK* siRNA group. By contrast, the *MERTK* siRNA + *CCT5*-Le group exhibited a significantly increased CCT5 expression and quantity of red particles phagocytized into HsRPE cells compared with the *MERTK* siRNA group (Figure 4A). To investigate whether *CCT5*-Le rescued the phagocytic function *via* the regulation of F-actin, the expression of F-actin was then examined. Our results indicate that *MERTK* siRNA significantly decreased the expression of F-actin at both the mRNA and protein levels, as compared with that in the NC group. In the *MERTK* siRNA + *CCT5* siRNA group, the expression of F-actin was decreased further compared with that in the *MERTK* siRNA group. However, in the *MERTK* siRNA + *CCT5*-Le group, F-actin expression was significantly increased, compared with that in the *MERTK* siRNA and *MERTK* siRNA + *CCT5* siRNA groups (Figures 4B,C). Furthermore, to detect whether *CCT5*-Le restored the distribution and arrangement of F-actin, we performed the immunofluorescence analysis. In the *MERTK* siRNA + *CCT5* siRNA group, the distribution and arrangement of F-actin became more irregular than that in the *MERTK* siRNA group. However, in the *MERTK* siRNA + *CCT5*-Le group, the distribution and arrangement of F-actin were recovered, as seen in the NC group (Figure 4D). These results indicate that the upregulation of CCT5 expression rescued the phagocytic function, which was disrupted by *MERTK* siRNA, by restoring the expression and organization of F-actin in HsRPE cells.

### Chaperonin-Containing TCP1 Subunit 5 Rescued Human Primary RPE Cells With Mer Receptor Tyrosine Kinase-Associated Retinitis Pigmentosa via the LIM-Kinase 1/Cofilin, but Not the SSH1/Cofilin, Pathway

**FIGURE 5:**
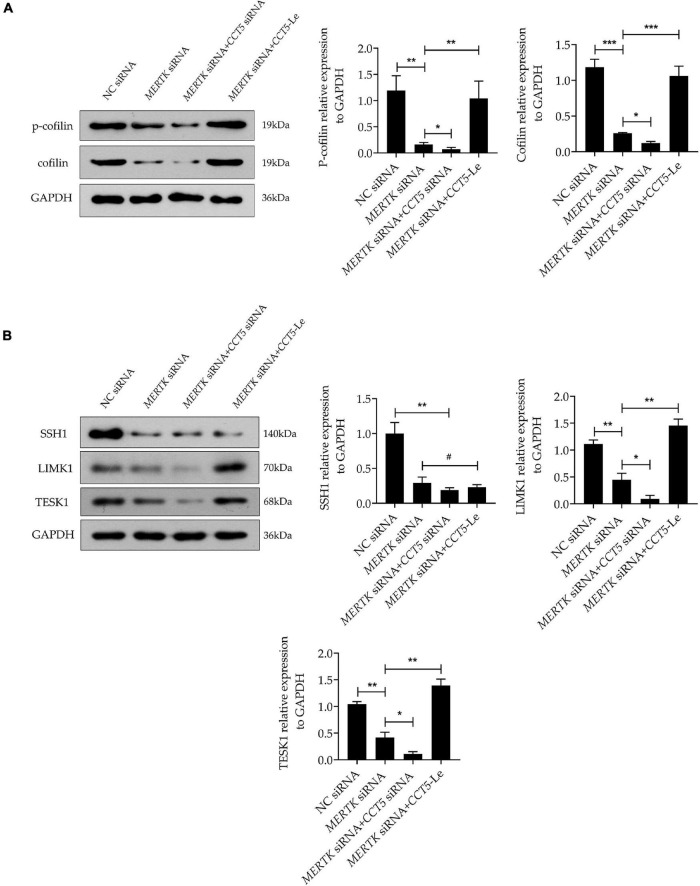
CCT5 rescued HsRPE cells with MERTK-associated RP *via* the LIMK1/cofilin, but not the SSH1/cofilin, pathway. **(A)** HsRPE cells were treated with or without *CCT5* siRNA or *CCT5*-Le after exposure to NC or *MERTK* siRNA for 48 h, and the cofilin and p-cofilin expression was analyzed by Western blotting. **(B)** HsRPE cells were treated the same as in **(A)**, and the expression of LIMK1, TESK1, and SSH1 was analyzed by Western blotting. *P < 0.05; **P < 0.01; *** *P* < 0.001.

Phosphorylated cofilin (p-cofilin) and cofilin are essential regulators of F-actin dynamics, regulating the polymerization and depolymerization of F-actin in the processes of phagocytosis and migration. To determine whether cofilin and p-cofilin were involved in *CCT5*-Le’s rescue of the phagocytosis and migration function of HsRPE cells with MERTK-associated RP, we next determined their levels of expression. Notably, we found that the expression of both cofilin and p-cofilin was suppressed in the *MERTK* siRNA group compared with that in the NC group. In the *MERTK* siRNA + *CCT5* siRNA group, the expression of both cofilin and p-cofilin was decreased further when compared with that in the *MERTK* siRNA group. However, in the *MERTK* siRNA + *CCT5*-Le group, the expression of both cofilin and p-cofilin was increased compared with that in the *MERTK* siRNA and *MERTK* siRNA + *CCT5* siRNA groups (Figure 5A). Cofilin is inactivated *via* phosphorylation by LIM-kinase 1 (LIMK1) and testicular protein kinase 1 (TESK1) and reactivated *via* dephosphorylation by slingshot 1 (SSH1). Therefore, to investigate whether LIMK1, TESK1, and SSH1 regulated the activity of cofilin in the process of *CCT5*-Le’s rescue of the phagocytosis and migration function of HsRPE cells under MERTK-associated RP, we detected the expression of LIMK1, TESK1, and SSH1. The results indicate that the expression of LIMK1, TESK1, and SSH1 was decreased in the *MERTK* siRNA group, compared with that in the NC group. However, in the *MERTK* siRNA + *CCT5* siRNA group, only the expression of LIMK1 and TESK1, and not SSH1, was further decreased compared with that in the *MERTK* siRNA group. In the *MERTK* siRNA + *CCT5*-Le group, the expression of LIMK1 and TESK1 was increased, but the expression of SSH1 again demonstrated no significant change compared with that in the *MERTK* siRNA and *MERTK* siRNA + *CCT5* siRNA groups (Figure 5B). These results indicate that *CCT5*-Le rescued the phagocytosis and migration function which were destroyed by *MERTK* siRNA *via* the LIMK1/cofilin, but not the SSH1/cofilin, pathway.

## Discussion

Retinitis pigmentosa is caused by multiple molecular interactions, of which the phagocytic dysfunction induced by a *MERTK* mutation in RPE cells is one of the most vital (1). As a cytoskeleton actin protein, F-actin plays an important role in cellular motility and phagocytosis (27). CCT5 acts as a chaperone protein and plays a vital role in maintaining the normal function and structure of actin proteins (28). Recently, numerous studies have demonstrated the role that F-actin plays in the phagocytosis of RPE cells (29). However, to date, studies have failed to determine whether CCT5 is associated with F-actin regulation or whether it plays an essential role in the progression of RP. Therefore, in this study, we focused on the role of CCT5’s regulation of F-actin in the pathogenesis of RP and the molecular mechanism of such.

**FIGURE 6:**
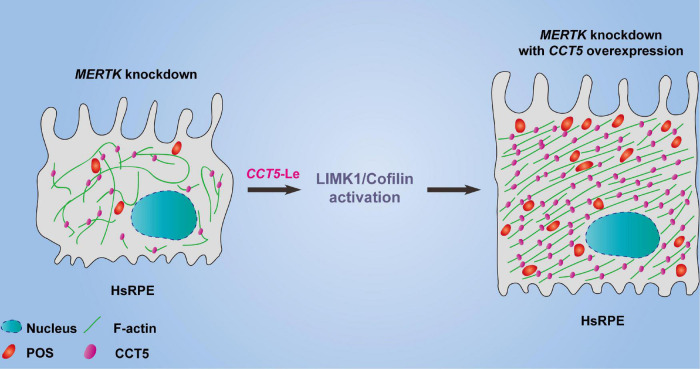
The model diagram of *CCT5*-specific lentiviral vectors (*CCT5*-Le) rescued the cell phagocytic function of HsRPE cells under *MERTK* siRNA condition by increasing the expression of F-actin and restoring its regular arrangement *via* the LIMK1/cofilin pathway.

We found that downregulating MERTK inhibited cell proliferation, migration, and phagocytic function. Additionally, knocking down MERTK significantly inhibited F-actin’s expression and disrupted its regular arrangement. Importantly, our study revealed, for the first time, that CCT5 interacts with F-actin and that CCT5 expression is inhibited by *MERTK* siRNA in HsRPE cells. Upregulating CCT5 rescued the cell proliferation, migration, and phagocytic function which were destroyed by *MERTK* siRNA *via* an increase in the expression of F-actin, as well as the remodeling of its regular arrangement. Moreover, we discovered that CCT5 promoted the remodeling of F-actin’s arrangement *via* the LIMK1/cofilin, but not the SSH1/cofilin, pathway (Figure 6).

Phagocytosis in RPE cells is a highly conserved, complex process that has evolved to counter the constant shedding of POS (30). To maintain the healthy photoreceptor–RPE interface of the retina, RPE cells constantly phagocytize the POS; however, the mutation of MERTK results in phagocytic dysfunction in RPE cells, leading to RP. However, which part of the phagocytic process is disrupted in RPE cells with the MERTK mutation remains to be elucidated. Ensheathment is required for POS fragmentation before internalization. The MERTK ligands Gas6 and Protein S contribute to the initiation of POS ensheathed by RPE (31). Abnormalities in the ensheathment, fragmentation, and internalization process in POS phagocytosis were found to occur in MERTK-mutated RPE while restoring MERTK expression in the RPE cells of RP patients reversed these abnormalities (32). The ensheathment, fragmentation, and internalization process require an appropriate F-actin distribution and arrangement (33). Therefore, the appropriate expression and correct spatial organization of F-actin filaments are essential for normal proliferation, migration, and phagocytic function (34–36). A recent study reported that, due to the lack of Mertk, the RPE cells of RCS rats could not aggregate F-actin and bind POS, even in the presence of protein S (14). Additionally, a previous study revealed that hypophagocytic RPE cells contain F-actin stress fibers but lack adjacent lateral circumferential F-actin (13). Together, these studies indicate that F-actin is involved in MERTK-associated RP. In line with these findings, our MERTK-associated RP model in HsRPE cells demonstrated that knocking down MERTK significantly inhibited the expression of F-actin and disrupted its regular arrangement. Moreover, we found that downregulating MERTK expression inhibited proliferation, migration, and phagocytic function.

Filamentous actin is regulated by various actin-binding proteins (37). Cofilin is a ubiquitous actin-binding protein that binds to F-actin only in its dephosphorylated state. When dephosphorylated cofilin binds to actin, it depolymerizes F-actin; however, phosphorylation at Ser 3 inactivates cofilin which then separates from, and thereby repolymerizes, F-actin (38). The recombination of the actin cytoskeleton *via* polymerization and depolymerization of F-actin by cofilin is indispensable for various cellular activities, such as phagocytosis, cytokinesis, and cell migration (39). The balance between F-actin and cofilin is vital for preserving the normal physiological function of cells. Once this balance is disturbed, many diseases can result—including Alzheimer’s disease (40, 41), mitotic disorder (42), cancers (43), optic nerve injury (44), migration disorder (35), immune dysfunction (45), and more. However, whether cofilin takes part in MERTK-associated RP retains a veil of mystery. First, we revealed that knocking down MERTK significantly inhibited the expression of both cofilin and p-cofilin in HsRPE cells. These results are consistent with the abnormal expression and arrangement of F-actin in our MERTK-associated RP model in HsRPE cells. The phosphorylation of cofilin is controlled by the LIMK1/TESK1 pathway (46–48). LIMK1 and TESK1 are, therefore, two key components of the signal transduction pathways that link extracellular stimulation to changes in cytoskeletal structure (49, 50). It was reported that the N-terminal kinase of TESK1 may be a putative candidate accounting for LIMK-independent cofilin phosphorylation, as it shows approximately 50% amino-acid identity with LIMK (51, 52). On the other hand, cofilin is activated by dephosphorylation by the phosphatase SSH1. SSH1’s activity is strongly increased by its binding to F-actin (53). According to previous studies, the overexpression of LIMK1 and TESK1 in cultured cells results in the accumulation of F-actin, and the overexpression of SSH1 leads to the depolymerization of F-actin (49, 50, 53). However, no currently available research indicates a regulatory relationship between LIMK1, TESK1, SSH1, cofilin, and F-actin in RP. In our study, we revealed that the expression of LIMK1, TESK1, and SSH1 was inhibited after MERTK expression was downregulated. These results explain why knocking down MERTK significantly inhibited the expression of both cofilin and p-cofilin in HsRPE cells.

As a chaperonin, CCT5 assists in the folding of the unfolding or misfolded proteins, and actin is reported to be a natural substrate for CCT5 (21). It is reported that the effect of CCT5 on the z-disk of sarcomere cells is required for the efficient assembly of actin filaments, and CCT5-specific ATP binding is required for the efficient folding of actin *in vivo*. In addition, the mutant A-actin subtype that causes linear myopathy in patients acquires its pathogenic conformation through this function of CCT5 (22). Julie et al. found that CCT depletion did not affect actin-polypeptide synthesis but led to a decrease in natural actin levels and a disturbance of actin-based cell motility (54). McCormack et al. found that actin appeared to fold in association with chaperonin and that actin site II—located at the apex of actin subdomain 4—was the main binding site for CCT binding (55). Llorca et al. produced a three-dimensional reconstruction of the CCT and α-actin complex, which demonstrated that α-actin interacts with the apical domain of one of the two CCT subunits. Furthermore, they found that actin’s binding to CCT is subunit-specific and geometrically dependent (21). CCT5’s interactions in HsRPE cells with the MERTK mutation have not yet been reported. In our study, we discovered that CCT5 interacted with F-actin and that CCT5’s expression was inhibited by *MERTK* siRNA in HsRPE cells. To verify that F-actin was regulated by CCT5, we downregulated and upregulated CCT5 separately in HsRPE cells. We found that upregulating the expression of CCT5 using *CCT5*-Le increased the expression of F-actin and remodeled its distribution and arrangement in our MERTK-associated RP model in HsRPE cells. When the expression of CCT5 was decreased using CCT siRNA, the effect was exactly opposite to that of *CCT5*-Le. In addition to its effect on actin, CCT5 has been reported to be connected to multiple cellular processes including proliferation, migration, and apoptosis *via* its relation with numerous proteins such as cyclin D1, polymerase basic protein, cell-division cycle protein 20, and P53 (56–59). In our study, we found that CCT5 regulated the expression of LIMK1 and TESK1 but not SSH1. Moreover, upregulating CCT5 rescued the cell proliferation, migration, and phagocytic function of HsRPE cells under MERTK-associated RP. These results indicate that CCT5 protects against MERTK-associated RP in RPE cells through interactions with F-actin and the activation of the LIMK1/cofilin pathway.

However, there are several limitations to our study. First, we only performed *in vitro* experiments in an RPE cell model established using *MERTK* siRNA. *In vivo* experiments should be performed in RCS rats to verify the role of CCT5 in MERTK-associated RP. Many questions remain to be answered in RCS rats, including whether the expression of CCT5 is abnormal in the RPE cells of RCS rats and whether regulating the expression of CCT5 can rescue the RP. Second, although CCT consists of eight subunits, we only detected the function of CCT5 in HsRPE cells. Thus, whether other paralogous subunits are involved in RP requires further exploration. Third, we found that the expression of cofilin, p-cofilin, LIMK1, and TESK1 was upregulated by CCT5-Le, but how CCT5 increases their expression—whether by increasing their transcription or by decreasing their degradation—remains to be determined. Although LIMK1 and TESK1 control the phosphorylation of cofilin, no studies have demonstrated that they control the expression of cofilin. Therefore, whether CCT5 directly increases total cofilin expression or indirectly increases cofilin levels *via* other signals requires further study. We will attempt to find answers to these questions in future research.

In summary, despite the abovementioned limitations, our results demonstrate, for the first time, that the cell proliferation, migration, and phagocytic function of RPE cells are inhibited by MERTK-associated RP due to the abnormal expression of F-actin and disruption of its regular arrangement. Importantly, our findings reveal that CCT5 interacts with F-actin and regulates its expression and arrangement. Upregulating CCT5 protects against the effect of *MERTK* knockdown in RPE cells through its interactions with F-actin and activation of the LIMK1/cofilin pathway. These findings provide a new perspective for research into the mechanisms underlying MERTK-associated RP and provide a new direction for future studies of the molecular mechanisms of RP pathogenesis.

## Materials and Methods

### Cell Culture

Human donor eyes were obtained from the Guangdong Eye Bank in accordance with the 2013 Declaration of Helsinki. Written consent was obtained from the donor or the donor’s family for the eye to be used in medical research. The study was approved by the Ethics Committee of Zhongshan Eye Center, Sun Yat-sen University. The human primary RPE (HsRPE) cells were separated from human donor eyes as previously described (60). All the HsRPE cells were cultured in DMEM containing 4.5 g/L glucose, which was supplemented with 1% penicillin/streptomycin and 10% FBS, in a humidified incubator with a 5% CO2 atmosphere at 37°C.

### siRNA Transfections

The siRNAs for *MERTK* and *CCT5* were designed and synthesized by RiboBio Inc. (Guangzhou, China). The sequences of the *MERTK* siRNA and *CCT5* siRNA are listed in Table 1. Transient transfections of the siRNAs for human *MERTK* (20 nM) and *CCT5* (20 nM) were conducted using Lipofectamine RNAiMAX (Invitrogen, New York, NY, United States), according to the manufacturer’s protocol. Morphological changes in the cells were investigated using an inverted microscope (Carle ZEISS Axio Observer 7, Oberkohen, Baden-wurttemberg, Germany), 48 h after siRNA transfection.

**TABLE 1:** List of siRNA sequences.

Gene	Sequences (5′–3′)
*MERTK* siRNA1	GGATGAAGCCTCCGACTAA
*MERTK* siRNA2	GGTGACCTCTGTCGAATCA
*CCT5* siRNA1	GGAGAGACGTTGACTTTGA
*CCT5* siRNA2	GCGATAGCGTCCTTGTTGA
*NC* siRNA	TTCTCCGAACGTGTCACGT

### Real-Time PCR

The RNA of the HsRPE samples was extracted using Trizol reagent (Invitrogen, New York, NY, United States). After cDNA was synthesized by reverse transcribing the RNA, the RT-PCR assay was performed using a LightCycler 480 SYBR Green I Master (Roche, Indianapolis, IN, United States). The values for each gene were normalized to the levels of tubulin or 18S mRNA. The sequences of the primers used for RT-PCR are listed in Table 2.

**TABLE 2:** Sequences of PCR primers.

Gene	Forward sequence (5′-3′)	Reverse sequence (5′-3′)
*MERTK*	GGAAATAGCTACGCGGGGAA	TTCCGAACGTCAGGCAAACT
*CCT5*	AGTATGCCATGAGAGCGTTT	GCAGGGTTCATCTCCTTCAC
*F-actin*	TGATGTTAGGCCTGCAAGA	GTAGTGCTGCATCAATTTCC
*18s*	GTAACCCGTTGAACCCCA	CCATCCAATCGGTAGTAG

### Western Blot Assay

Cell lysis buffer was used to extract the total protein of the HsRPE cell samples before it was separated by sodium dodecyl sulfate polyacrylamide gel electrophoresis (SDS-PAGE). The total protein was then transferred onto polyvinylidene difluoride (PVDF) membranes (Millipore, Bedford, MA, United States). The PVDF membranes were incubated with different primary antibodies: anti-MERTK (Cell Signaling Technology, 4319S), anti-F-actin (Abcam, ab130935), anti-CCT5 (Proteintech, 11603-1-AP), anti-cofilin (Abcam, ab54532), anti-phosphorylated cofilin (p-cofilin, Abcam, ab12866), anti-LIMK1 (Affinity, AF6345), anti-TESK1 (Affinity, DF4012), anti-SSH1 (Abcam, ab76943), and anti-GAPDH (Abcam, ab8245). The membranes were incubated with the corresponding secondary antibody for 1 h at room temperature. The binding of specific antibodies was visualized using Chemiluminescent fluid (Millipore, WBKLS0500).

### Cell Counting Kit-8 Assay

A CCK8 kit (Beyotime, Shanghai, China) was used to determine the cell viability. The HsRPE cells were cultured at a density of 7 × 10^3^ cells/well in 96-well plates, after exposure to MERTK siRNA with or without the interference of CCT5 expression for 48 h. The CCT8 assay was performed as previously described in Zhou et al. (61), and the absorption values at a wavelength of 570 nm were evaluated.

### Wound-Healing and Transwell-Migration Assays

Wound-healing assays were performed as previously described in Qi et al. (62). Briefly, HsRPE cells from all the groups were seeded into 24-well plates until 100% confluence was attained. Wounds were scratched in the confluent cell layers using 200 uL pipette tips, and the samples were cultured for a further 24 h. The Transwell assays were performed in 24-well plates with a chamber insert (8 μm pore size) (Corning, 3422, Corning, NY, United States). After 24 h of incubation with transfected *MERTK* siRNAs, *CCT5* siRNAs, or *CCT5* lentiviral vectors (*CCT5*-Le), the underlying HsRPE cells were stained with crystal violet before the number of migrated cells was determined using an inverted microscope (Carle ZEISS Axio Observer 7, Oberkohen, Baden-wurttemberg, German).

### Phagocytosis Assay

Phagocytosis assays were performed as previously described in Irschick et al. (63). To assess the phagocytosis of HsRPE cells, Nile red-labeled Fluospheres (Sigma-Aldrich, F8825) were used. HsRPE cells from all the groups were cultured until the cell confluence reached 80%. Then, the medium was replaced and 10 μL/mL of diluted 2 μm-diameter particles were added. HsRPE cells were phagocytized with microspheres in a 5% CO2 atmosphere at 37°C for 6 h and then washed with PBS three times to remove the unphagocytized particles before being stained with 4′,6-diamidino-2-phenylindole (DAPI) for 10 min. Finally, the number of microspheres was quantified by measuring the total fluorescence present using a confocal microscope (Carle ZEISS Axio Imager Z2, Oberkohen, Germany).

### Immunofluorescence

The immunofluorescence assays were performed as previously described in Feng et al. (64). HsRPE cells were fixed with 4% paraformaldehyde for 15 min, followed by blocking with 5% BSA at room temperature for 1 h. The cell samples were incubated with RPE65 (Abcam, ab235950), F-actin (Abcam, ab130935), CCT5 (Proteintech, 11603-1-AP), and MERTK (Cell Signaling Technology, 4319S) antibodies. Cell samples were then incubated with secondary antibodies for 1 h at room temperature. Subsequently, the nuclei of the cells were stained using DAPI for 7 min. All the images were taken using a confocal microscope (Carle ZEISS LSM 980, Oberkohen, Germany).

### Co-immunoprecipitation

The total protein of the HsRPE cell samples was extracted using cell lysis buffer, leaving 100 μL of cell lysate as the input group. Two 1.5 EP tubes were filled with 400 μL of HsPRE cell lysis solution, and 100 uL of RIPA lysis buffer was added to each. One tube was incubated under rotation with 5 μg of anti-CCT5 (Proteintech, 11603-1-AP), while the other tube was incubated under rotation with 5 μg of rabbit anti-IgG (Cell Signaling Technology, 2729) at 4°C overnight. Fifty microliters of Protein A/G Magnetic Beads (Bimake Ferromagnetic Bead, B23202) were added to two additional 1.5 mL EP tubes. The magnetic beads were washed twice with 1 ml of precooled PBS and centrifuged at 3000 rpm for 1 min. Next, the magnetic beads were rotated and blocked with 3% BSA at 4°C for 30 min, followed by centrifugation at 3000 rpm for 1 min, absorbing the blocking solution, adding the antibody-protein complexes into the magnetic beads, and incubating under rotation at room temperature for 1 h. After incubation, the magnetic beads were washed with washing buffer. After the centrifugation of the proteins at 12,000 rpm and 4°C for 1 min, the supernatant was discarded carefully. Next, the proteins were separated by SDS-PAGE and transferred onto PVDF membranes. The membranes were probed with anti-F-actin (Abcam, ab130935) and anti-CCT5 (Proteintech, 11603-1-AP), and their corresponding secondary antibodies. Specific antibody binding was visualized using chemiluminescent fluid (Millipore, WBKLS0500).

### Transfection and Expression of Chaperonin-Containing TCP1 Subunit 5-Le

Lentiviral vectors were used to upregulate the expression of CCT5. The recombined plasmid, contained pLV-CMV.hCCT5.EF1.CopGFP-T2A-Puro.Wpre, was used to produce the lentivirus. The 293 T cells were then transfected with the lentivirus. After 72 h, the 293 T cells were collected, the concentrated virus was extracted by centrifugation, and the virus titer was detected. The HsRPE cells were then inoculated in 6-well plates and incubated until their confluence was about 30%, and 50 μL of virus was added (titer: 2 × 10^8^ TU/mL). After another 48 h of culture, the expression of the target gene in the lentivirus was observed by measuring the green fluorescent protein (GFP), and the mRNA and protein levels were detected. The sequences of the lentiviral vectors of CCT5 are listed in Table 3.

**TABLE 3:** Sequences of the lentiviral vectors of CCT5.

Name	Sequence
Lentiviral vectors of *CCT5*	ATGAACTCCTCCTTGGGACCCACTATCGAGAAACTATCAGTGTCTCATATAATGGCAGCAAAGGCTGTAGCAAATACAATGAGAACAT CACTTGGACCAAATGGGCTTGATAAGATGATGGTGGATAAGGATGGAGATGTGACTGTAACTAATGATGGGGCCACCATCTTAAGCA TGATGGATGTTGATCATCAGATTGCCAAGCTGATGGTGGAACTGTCCAAGTCTCAGGATGATGAAATTGGAGATGGAACCACAGGAG TGGTTGTCCTGGCTGGTGCCTTGTTAGAAGAAGCGGAGCAATTGCTAGACCGAGGCATTCACCCAATCAGAATAGCCGATGGCTAT GAGCAGGCTGCTCGTGTTGCTATTGAACACCTGGACAAGATCAGCGATAGCGTCCTTGTTGACATAAAGGACACCGAACCCCTGAT TCAGACAGCAAAAACCACGCTGGGCTCCAAAGTGGTCAACAGTTGTCACCGACAGATGGCTGAGATTGCTGTGAATGCCGTCCTC ACTGTAGCAGATATGGAGCGGAGAGACGTTGACTTTGAGCTTATCAAAGTAGAAGGCAAAGTGGGCGGCAGGCTGGAGGACACTA AACTGATTAAGGGCGTGATTGTGGACAAGGATTTCAGTCACCCACAGATGCCAAAAAAAGTGGAAGATGCGAAGATTGCAATTCTC ACATGTCCATTTGAACCACCCAAACCAAAAACAAAGCATAAGCTGGATGTGACCTCTGTCGAAGATTATAAAGCCCTTCAGAAATAC GAAAAGGAGAAATTTGAAGAGATGATTCAACAAATTAAAGAGACTGGTGCTAACCTAGCAATTTGTCAGTGGGGCTTTGATGATGAA GCAAATCACTTACTTCTTCAGAACAACTTGCCTGCGGTTCGCTGGGTAGGAGGACCTGAAATTGAGCTGATTGCCATCGCAACAGG AGGGCGGATCGTCCCCAGGTTCTCAGAGCTCACAGCCGAGAAGCTGGGCTTTGCTGGTCTTGTACAGGAGATCTCATTTGGGACA ACTAAGGATAAAATGCTGGTCATCGAGCAGTGTAAGAACTCCAGAGCTGTAACCATTTTTATTAGAGGAGGAAATAAGATGATCATTGA GGAGGCGAAACGATCCCTTCACGATGCTTTGTGTGTCATCCGGAACCTCATCCGCGATAATCGTGTGGTGTATGGAGGAGGGGCT GCTGAGATATCCTGTGCCCTGGCAGTTAGCCAAGAGGCGGATAAGTGCCCCACCTTAGAACAGTATGCCATGAGAGCGTTTGCCG ACGCACTGGAGGTCATCCCCATGGCCCTCTCTGAAAACAGTGGCATGAATCCCATCCAGACTATGACCGAAGTCCGAGCCAGACA GGTGAAGGAGATGAACCCTGCTCTTGGCATCGACTGTTTGCACAAGGGGACAAATGATATGAAGCAACAGCATGTCATAGAAACCT TGATTGGCAAAAAGCAACAGATATCTCTTGCAACACAAATGGTTAGAATGATTTTGAAGATTGATGACATTCGTAAGCCTGGAGAATCT GAAGAATGA

### Statistical Analysis

Statistical analysis was performed using SPSS 26.0. An unpaired *t*-test and one-way ANOVA were used to analyze and compare the differences between groups. The data are expressed as the means ± standard deviations (SD) of at least three independent trials. A *p*-value less than 0.05 was considered statistically significant.

## Data Availability Statement

The raw data supporting the conclusions of this article will be made available by the authors, without undue reservation.

## Ethics Statement

The studies involving human participants were reviewed and approved by the Ethics Committee of Zhongshan Eye Center, Sun Yat-sen University. The patients/participants provided their written informed consent to participate in this study.

## Author Contributions

LF designed the experiments and wrote the manuscript. HL performed the experiments. YD performed the literature search and provided experimental support. TZ, YtZ, ZdL, LZ, and XW collected the data. GW, LbZ, ZJ, and ZL analyzed the data. ZO and YW performed the extraction of HsRPE cells. YhZ supervised the project and reviewed the manuscript. LF, HL, and YD have made equal contributions to the study and should therefore be regarded as equal authors. All authors have read and approved the published version of the manuscript.

## Conflict of Interest

The authors declare that the research was conducted in the absence of any commercial or financial relationships that could be construed as a potential conflict of interest.

## Publisher’s Note

All claims expressed in this article are solely those of the authors and do not necessarily represent those of their affiliated organizations, or those of the publisher, the editors and the reviewers. Any product that may be evaluated in this article, or claim that may be made by its manufacturer, is not guaranteed or endorsed by the publisher.

## Abbreviations

CCK8: Cell Counting Kit-8
CCT: chaperonin-containing TCP-1
CCT5: chaperonin-containing TCP1 subunit 5
CCT5-Le: CCT5-specific lentiviral vectors
Co-IP: co-immunoprecipitation
F-actin: filamentous actin
HsRPE: human primary RPE
LIMK1: LIM-kinase 1
MERTK: Mer receptor tyrosine kinase
NC: negative control
OD: optical density
p-cofilin: Phosphorylated cofilin
POS: photoreceptor outer segment
RCS: Royal College of Surgeons
RP: retinitis pigmentosa
RPE: retinal pigment epithelium
RT-PCR: quantitative real-time PCR
SSH1: slingshot 1
TESK1: testicular protein kinase 1.

## References

[1] Hartong DT Berson EL Dryja TP. Retinitis pigmentosa. *Lancet.* (2006) 368:1795–809. 10.1016/s0140-6736(06)69740-7 17113430

[2] Humphries P Kenna P Farrar GJ. On the molecular genetics of retinitis pigmentosa. *Science.* (1992) 256:804–8. 10.1126/science.1589761 1589761

[3] Dias MF Joo K Kemp JA Fialho S Cunha ADS Woo SJ Molecular genetics and emerging therapies for retinitis pigmentosa: basic research and clinical perspectives. *Prog Retin Eye Res.* (2018) 63:107–31. 10.1016/j.preteyeres.2017.10.004 29097191

[4] Ducloyer J-B Le Meur G Cronin T Adjali O Weber M. Gene therapy for retinitis pigmentosa. *Med Sci.* (2020) 36:607–15. 10.1051/medsci/2020095 32614312

[5] Gal A Li Y Thompson D Weir J Orth U Jacobson S Mutations in MERTK, the human orthologue of the RCS rat retinal dystrophy gene, cause retinitis pigmentosa. *Nat Genet.* (2000) 26:270–1. 10.1038/81555 11062461

[6] D’Cruz PM Yasumura D Weir J Matthes MT Abderrahim H Lavail MM Mutation of the receptor tyrosine kinase gene MERTK in the retinal dystrophic RCS rat. *Hum Mol Genet.* (2000) 9:645–51. 10.1093/hmg/9.4.645 10699188

[7] Kwon W Freeman SA. Phagocytosis by the retinal pigment epithelium: recognition, resolution, recycling. *Front Immunol.* (2020) 11:604205. 10.3389/fimmu.2020.604205 33281830 PMC7691529

[8] Essner E Pino RM Griewski RA. Distribution of anionic sites on the surface of retinal pigment epithelial and rod photo-receptor cells. *Curr Eye Res.* (1981) 1:381–9. 10.3109/02713688109019975 7318491

[9] Bulloj A Duan W Finnemann SC. PI 3-kinase independent role for AKT in F-actin regulation during outer segment phagocytosis by RPE cells. *Exp Eye Res.* (2013) 113:9–18. 10.1016/j.exer.2013.05.002 23669303 PMC3737417

[10] Finnemann SC Rodriguez-Boulan E. Macrophage and retinal pigment epithelium phagocytosis: apoptotic cells and photoreceptors compete for alphavbeta3 and alphavbeta5 integrins, and protein kinase C regulates alphavbeta5 binding and cytoskeletal linkage. *J Exp Med.* (1999) 190:861–74. 10.1084/jem.190.6.861 10499924 PMC2195631

[11] Bulloj A Maminishkis A Mizui M Finnemann SC. Semaphorin4D-PlexinB1 Signaling Attenuates Photoreceptor Outer Segment Phagocytosis by Reducing Rac1 Activity of RPE Cells. *Mol Neurobiol.* (2018) 55:4320–32. 10.1007/s12035-017-0649-5 28624895 PMC5733634

[12] Freeman SA Goyette J Furuya W Woods EC Bertozzi CR Bergmeier W Integrins form an expanding diffusional barrier that coordinates phagocytosis. *Cell.* (2016) 164:128–40. 10.1016/j.cell.2015.11.048 26771488 PMC4715264

[13] Müller C Charniga C Temple S Finnemann SC. Quantified F-actin morphology is predictive of phagocytic capacity of stem cell-derived retinal pigment epithelium. *Stem Cell Rep.* (2018) 10:1075–87. 10.1016/j.stemcr.2018.01.017 29456184 PMC5918243

[14] Mao Y Finnemann S. Acute RhoA/Rho kinase inhibition is sufficient to restore phagocytic capacity to retinal pigment epithelium lacking the engulfment receptor MERTK. *Cells.* (2021) 10:1927. 10.3390/cells10081927 34440696 PMC8394172

[15] Frydman J Nimmesgern E Erdjument-Bromage H Wall JS Tempst P Hartl FU. Function in protein folding of TRiC, a cytosolic ring complex containing TCP-1 and structurally related subunits. *Embo J.* (1992) 11:4767–78. 10.1002/j.1460-2075.1992.tb05582.x 1361170 PMC556952

[16] Cong Y Baker ML Jakana J Woolford D Miller EJ Reissmann S 4.0-A resolution cryo-EM structure of the mammalian chaperonin TRiC/CCT reveals its unique subunit arrangement. *Proc Natl Acad Sci USA.* (2010) 107:4967–72. 10.1073/pnas.0913774107 20194787 PMC2841888

[17] Rommelaere H Van Troys M Gao Y Melki R Cowan NJ Vandekerckhove J Eukaryotic cytosolic chaperonin contains t-complex polypeptide 1 and seven related subunits. *Proc Natl Acad Sci USA.* (1993) 90:11975–9. 10.1073/pnas.90.24.11975 7903455 PMC48108

[18] Frydman J Hartl FU. Principles of chaperone-assisted protein folding: differences between in vitro and in vivo mechanisms. *Science.* (1996) 272:1497–502. 10.1126/science.272.5267.1497 8633246

[19] Dekker C Stirling P McCormack EA Filmore H Paul A Brost RL The interaction network of the chaperonin CCT. *Embo J.* (2008) 27:1827–39. 10.1038/emboj.2008.108 18511909 PMC2486426

[20] Llorca O Martín-Benito J Grantham J Ritco-Vonsovici M Willison KR Carrascosa JL The ‘sequential allosteric ring’ mechanism in the eukaryotic chaperonin-assisted folding of actin and tubulin. *Embo J.* (2001) 20:4065–75. 10.1093/emboj/20.15.4065 11483510 PMC149171

[21] Llorca O McCormack EA Hynes GM Grantham JJ Cordell J Carrascosa JL Eukaryotic type II chaperonin CCT interacts with actin through specific subunits. *Nature.* (1999) 402:693–6. 10.1038/45294 10604479

[22] Berger J Berger S Li M Jacoby AS Arner A Bavi N In vivo function of the chaperonin TRiC in α-actin folding during sarcomere assembly. *Cell Rep.* (2018) 22:313–22. 10.1016/j.celrep.2017.12.069 29320728

[23] Sergeeva O Tran MT Haase-Pettingell C King JA. Biochemical characterization of mutants in chaperonin proteins CCT4 and CCT5 associated with hereditary sensory neuropathy. *J Biol Chem.* (2014) 289:27470–80. 10.1074/jbc.m114.576033 25124038 PMC4183788

[24] Pavel M Imarisio S Menzies FM Jimenez-Sanchez M Siddiqi FH Wu X CCT complex restricts neuropathogenic protein aggregation via autophagy. *Nat Commun.* (2016) 7:13821. 10.1038/ncomms13821 27929117 PMC5155164

[25] Levanova N Steinemann M Böhmer KE Schneider S Belyi Y Schlosser A Characterization of the glucosyltransferase activity of *Legionella pneumophila* effector SetA. *Naunyn-Schmiedeberg Arch Pharmacol.* (2018) 392:69–79. 10.1007/s00210-018-1562-9 30225797

[26] Ogneva IV Biryukov NS. Lecithin prevents cortical cytoskeleton reorganization in rat soleus muscle fibers under short-term gravitational disuse. *PLoS One.* (2016) 11:e0153650. 10.1371/journal.pone.0153650 27073851 PMC4830545

[27] Gruenheid S Finlay BB. Microbial pathogenesis and cytoskeletal function. *Nature.* (2003) 422:775–81. 10.1038/nature01603 12700772

[28] Mádi A Mikkat S Ringel B Thiesen HJ Glocker MO. Profiling stage-dependent changes of protein expression in *Caenorhabditis* elegans by mass spectrometric proteome analysis leads to the identification of stage-specific marker proteins. *Electrophoresis.* (2003) 24:1809–17. 10.1002/elps.200305390 12783458

[29] Czuczman MA Fattouh R van Rijn J Canadien V Osborne S Muise AM Listeria monocytogenes exploits efferocytosis to promote cell-to-cell spread. *Nature.* (2014) 509:230–4. 10.1038/nature13168 24739967 PMC4151619

[30] Kim J-Y Zhao H Martinez J Doggett TA Kolesnikov AV Tang PH Noncanonical Autophagy Promotes the Visual Cycle. *Cell.* (2013) 154:365–76. 10.1016/j.cell.2013.06.012 23870125 PMC3744125

[31] Nandrot EF. Opposite roles of MERTK ligands Gas6 and protein s during retinal phagocytosis. *Adv Exp Med Biol.* (2018) 1074:577–83. 10.1007/978-3-319-75402-4_70 29721990

[32] Almedawar S Vafia K Schreiter S Neumann K Khattak S Kurth T MERTK-dependent ensheathment of photoreceptor outer segments by human pluripotent stem cell-derived retinal pigment epithelium. *Stem Cell Rep.* (2020) 14:374–89. 10.1016/j.stemcr.2020.02.004 32160519 PMC7066375

[33] Hayes MJ Shao D Bailly M Moss SE. Regulation of actin dynamics by annexin 2. *Embo J.* (2006) 25:1816–26. 10.1038/sj.emboj.7601078 16601677 PMC1456940

[34] Haralambiev L Nitsch A Jacoby JM Strakeljahn S Bekeschus S Mustea A Cold atmospheric plasma treatment of chondrosarcoma cells affects proliferation and cell membrane permeability. *Int J Mol Sci.* (2020) 21:2291. 10.3390/ijms21072291 32225067 PMC7177321

[35] Bisaria A Hayer A Garbett D Cohen D Meyer T. Membrane-proximal F-actin restricts local membrane protrusions and directs cell migration. *Science.* (2020) 368:1205–10. 10.1126/science.aay7794 32527825 PMC8283920

[36] Kitano M Nakaya M Nakamura T Nagata S Matsuda M. Imaging of Rab5 activity identifies essential regulators for phagosome maturation. *Nature.* (2008) 453:241–5. 10.1038/nature06857 18385674

[37] McGough A. F-actin-binding proteins. *Curr Opin Struct Biol.* (1998) 8:166–76. 10.1016/s0959-440x(98)80034-1 9631289

[38] Hirayama A Adachi R Otani S Kasahara T Suzuki K. Cofilin plays a critical role in IL-8-dependent chemotaxis of neutrophilic HL-60 cells through changes in phosphorylation. *J Leukoc Biol.* (2006) 81:720–8. 10.1189/jlb.0506314 17130184

[39] Shiozaki N Nakano K Kushida Y Noguchi TQP Uyeda TQP Wloga D ADF/Cofilin Is Not Essential but Is Critically Important for Actin Activities during Phagocytosis in Tetrahymena thermophila. *Eukaryot Cell.* (2013) 12:1080–6. 10.1128/ec.00074-13 23729382 PMC3754538

[40] Bamburg JR Bernstein BW. Actin dynamics and cofilin-actin rods in alzheimer disease. *Cytoskeleton.* (2016) 73:477–97. 10.1002/cm.21282 26873625 PMC5345344

[41] Kang DE Woo JA. Cofilin, a master node regulating cytoskeletal pathogenesis in alzheimer’s disease. *J Alzheimer’s Dis.* (2019) 72:S131–44. 10.3233/JAD-190585 31594228 PMC6971827

[42] Jin Z Yao X Wen L Hao G Kwon J Hao J AIP1 and Cofilin control the actin dynamics to modulate the asymmetric division and cytokinesis in mouse oocytes. *Faseb J.* (2020) 34:11292–306. 10.1096/fj.202000093r 32602619

[43] Chang C-Y Leu J-D Lee Y-J. The actin depolymerizing factor (ADF)/cofilin signaling pathway and DNA damage responses in cancer. *Int J Mol Sci.* (2015) 16:4095–120. 10.3390/ijms16024095 25689427 PMC4346946

[44] Hao Q Zhang Y Li X Liang L Shi H Cui Z Upregulated neuregulin-1 protects against optic nerve injury by regulating the RhoA/cofilin/F-actin axis. *Life Sci.* (2020) 264:118283. 10.1016/j.lfs.2020.118283 32798561

[45] Burkhardt JK Carrizosa E Shaffer MH. The Actin Cytoskeleton in T Cell Activation. *Annu Rev Immunol.* (2008) 26:233–59. 10.1146/annurev.immunol.26.021607.090347 18304005

[46] Yang N Higuchi O Ohashi K Nagata K Wada A Kangawa K Cofilin phosphorylation by LIM-kinase 1 and its role in Rac-mediated actin reorganization. *Nature.* (1998) 393:809–12. 10.1038/31735 9655398

[47] Sakurai K Talukdar I Patil VS Dang J Li Z Chang K-Y Kinome-wide Functional Analysis Highlights the Role of Cytoskeletal Remodeling in Somatic Cell Reprogramming. *Cell Stem Cell.* (2014) 14:523–34. 10.1016/j.stem.2014.03.001 24702998 PMC4071169

[48] Røsok O Pedeutour F Ree AH Aasheim H-C. Identification and Characterization of TESK2, a Novel Member of the LIMK/TESK Family of Protein Kinases, Predominantly Expressed in Testis. *Genomics.* (1999) 61:44–54. 10.1006/geno.1999.5922 10512679

[49] Arber S Barbayannis FA Hanser H Schneider C Stanyon C Bernard O Regulation of actin dynamics through phosphorylation of cofilin by LIM-kinase. *Nat Cell Biol.* (1998) 393:805–9. 10.1038/31729 9655397

[50] Wang L Buckley AF Spurney RF. Regulation of cofilin phosphorylation in glomerular podocytes by testis specific kinase 1 (TESK1). *Sci Rep.* (2018) 8:12286. 10.1038/s41598-018-30115-3 30115939 PMC6095849

[51] Sarmiere PD Bamburg JR. Regulation of the neuronal actin cytoskeleton by ADF/cofilin. *J Neurobiol.* (2003) 58:103–17. 10.1002/neu.10267 14598374

[52] Yu Q Wu C Chen Y Li B Wang R Huang R Inhibition of LIM kinase reduces contraction and proliferation in bladder smooth muscle. *Acta Pharm Sin B.* (2021) 11:1914–30. 10.1016/j.apsb.2021.01.005 34386328 PMC8343115

[53] Eiseler T Döppler H Yan IK Kitatani K Mizuno K Storz P. Protein kinase D1 regulates cofilin-mediated F-actin reorganization and cell motility through slingshot. *Nat Cell Biol.* (2009) 11:545–56. 10.1038/ncb1861 19329994 PMC2761768

[54] Grantham J Brackley KI Willison KR. Substantial CCT activity is required for cell cycle progression and cytoskeletal organi-zation in mammalian cells. *Exp Cell Res.* (2006) 312:2309–24. 10.1016/j.yexcr.2006.03.028 16765944

[55] McCormack EA Rohman MJ Willison KR. Mutational screen identifies critical amino acid residues of beta-actin mediating interaction between its folding intermediates and eukaryotic cytosolic chaperonin CCT. *J Struct Biol.* (2001) 135:185–97. 10.1006/jsbi.2001.4389 11580268

[56] Meng Y Yang L Wei X Luo H Hu Y Tao X CCT5 interacts with cyclin D1 promoting lung adenocarcinoma cell migration and invasion. *Biochem Biophys Res Commun.* (2021) 567:222–9. 10.1016/j.bbrc.2021.04.105 34217974

[57] Zhang X Lin X Qin C Huang K Sun X Zhao L Avian Chaperonin Containing TCP1 Subunit 5 Supports Influenza A Virus Replication by Interacting With Viral Nucleoprotein, PB1, and PB2 Proteins. *Front Microbiol.* (2020) 11:538355. 10.3389/fmicb.2020.538355 33178142 PMC7593399

[58] Ooe A Kato K Noguchi S. Possible involvement of CCT5, RGS3, and YKT6 genes up-regulated in p53-mutated tumors in resistance to docetaxel in human breast cancers. *Breast Cancer Res Treat.* (2006) 101:305–15. 10.1007/s10549-006-9293-x 16821082

[59] Wang Q Huang W-R Chih W-Y Chuang K-P Chang C-D Wu Y Cdc20 and molecular chaperone CCT2 and CCT5 are required for the Muscovy duck reovirus p10.8-induced cell cycle arrest and apoptosis. *Veter Microbiol.* (2019) 235:151–63. 10.1016/j.vetmic.2019.06.017 31282373

[60] Terluk MR Ebeling MC Fisher CR Kapphahn RJ Yuan C Kartha RV N-Acetyl-L-cysteine Protects Human Retinal Pigment Epithelial Cells from Oxidative Damage: Implications for Age-Related Macular Degeneration. *Oxidative Med Cell Longev.* (2019) 2019:1–14. 10.1155/2019/5174957 31485293 PMC6710748

[61] Zhou J Jiang Y Chen H Wu Y Zhang L. Tanshinone I attenuates the malignant biological properties of ovarian cancer by inducing apoptosis and autophagy via the inactivation of PI3K/AKT/mTOR pathway. *Cell Prolif.* (2019) 53:e12739. 10.1111/cpr.12739 31820522 PMC7046305

[62] Qi T Jing R Wen C Hu C Wang Y Pei C Interleukin-6 promotes migration and extracellular matrix synthesis in retinal pigment epithelial cells. *Histochem Cell Biol.* (2020) 154:629–38. 10.1007/s00418-020-01923-4 32997263

[63] Irschick EU Sgonc R Böck G Wolf H Fuchs D Nussbaumer W Retinal pigment epithelial phagocytosis and metabolism differ from those of macrophages. *Ophthalmic Res.* (2004) 36:200–10. 10.1159/000078778 15292658

[64] Feng L Liang L Zhang S Yang J Yue Y Zhang X. HMGB1 downregulation in retinal pigment epithelial cells protects against diabetic retinopathy through the autophagy-lysosome pathway. *Autophagy.* (2021) 2021:1–20. 10.1080/15548627.2021.1926655 34024230 PMC8942416

